# Expanding patient access to psychiatry and mental health supports: early impacts of the Nova Scotia health rapid access and stabilization program

**DOI:** 10.3389/fpsyt.2025.1634335

**Published:** 2026-01-07

**Authors:** Medard K. Adu, Samuel Obeng Nkrumah, Belinda Agyapong, Ngozi Ezeanozie, Ejemai Eboreime, Gloria Obuobi-Donkor, Sanjana Sridharan, Jason Morrison, Bryanne Taylor, Monica MacKinnon, Mahmoud Awara, JianLi Wang, Cindy Feng, Wozney Lori, Prosper Koto, Vincent Israel Opoku Agyapong

**Affiliations:** 1 Department of Psychiatry, Faculty of Medicine, Dalhousie University, Halifax, NS, Canada; 2 Department of Psychiatry, Faculty of Medicine and Dentistry, University of Alberta, Edmonton, AB, Canada; 3 Mental Health and Addictions Program, Nova Scotia Health, Halifax, NS, Canada; 4 Department of Community Health and Epidemiology, Faculty of Medicine, Dalhousie University, Halifax, NS, Canada; 5 Research, Innovation and Discovery, Nova Scotia Health, Halifax, NS, Canada

**Keywords:** collaborative care model, early intervention, mental health care access, patient satisfaction, rapid access

## Abstract

**Background:**

The average Canadian waits several weeks to months to access a psychiatric consultation and other forms of mental health support even after referral by a primary healthcare provider (PHP). This delay can worsen conditions, leading to costly interventions like emergency department visits, unplanned hospital admissions, and extended inpatient care. To address these challenges, Nova Scotia Health launched the Rapid Access and Stabilization Program (RASP) in April 2023 at the QEII Health Sciences Centre in Halifax to enhance mental health care capacity and accessibility.

**Objectives:**

This study aims to evaluate the early impact of rapid access to psychiatry consultations compared to standard care and assess the service user experience of the RASP.

**Methods:**

This is an exploratory, descriptive study that utilizes retrospective secondary data to examine patient access to psychiatric consultations within the Mental Health and Addictions Program (MHAP) before RASP implementation (fiscal years 2021/2022 and 2022/2023) with data from the first year of RASP operation (fiscal year 2023/2024). A cross-sectional, survey-based approach was employed to assess patient satisfaction with RASP. Data on psychiatric consultations were obtained from provincial administrative sources, while patient satisfaction data were collected via the RASP satisfaction survey administered after consultations. Quantitative data were analyzed using descriptive and inferential statistics, and qualitative data from open-ended survey responses were manually analyzed using a content analysis approach.

**Results:**

In its first year, RASP provided psychiatric consultations to 960 unique patients. During the 2023/2024 fiscal year, there was a 1226.7% increase in PHP-referred patients receiving psychiatric assessments compared to 2022/2023. Additionally, the number of new patients assessed by a psychiatrist, either through PHP referral or transfer of care from mental health clinicians, increased by 102% compared to the previous fiscal year. A total of 487 participants responded to the satisfaction survey, with a mean overall satisfaction score of 9.31 (SD ± 1.42) on a scale from 1 (not satisfied) to 10 (best experience). Of the respondents, 80.5% indicated they would return for an assessment if needed, and 97.3% said they would recommend the program to others.

**Conclusion:**

This study reveals a substantial increase in access to psychiatric care through RASP and the program’s effectiveness is underscored by high overall satisfaction. These findings emphasize the program’s success in delivering quality mental health care while also identifying opportunities for further improvement to ensure continued patient satisfaction and service excellence.

## 1 Introduction

Mental disorders contribute to approximately one-third of global adult health-related disabilities, leading to significant individual suffering and economic burdens (1). Severe mental health issues, such as major depressive disorder, bipolar disorder, schizophrenia, and substance use disorders, impact individuals across all age brackets and are prevalent in all countries worldwide (2). Enhancing access to mental health care and reducing disparities in mental health care access is a pressing global need; however, current models of care and treatment strategies fall short of effectively addressing the global mental health care crisis (3). Despite increasing recognition of the importance of mental health, barriers to accessing timely and effective care persist (4). Among these barriers, long wait times for appointments, costs, uncertainty about where to seek help, insufficient funding, and inefficient service utilization have been identified as key obstacles to accessing care (5–7). Key barriers to accessing care include prolonged wait times for appointments, financial constraints, lack of clarity regarding where to seek assistance, inadequate funding, and inefficiencies in the utilization of services.

According to the Statistics Canada Report 2018, nearly 18% (5.3 million) of Canadians expressed a need for mental health support, and only slightly over half of them (56.2%; 3 million) reported having their needs fully met, while the remaining portion (43.8%; 2.3 million) indicated their needs were only partially met or unmet altogether, especially among those without a regular healthcare provider (8). Provincially, Nova Scotia aligns closely with these national trends, with the greatest unmet needs being related to access to counseling and psychiatric services (8, 9). This overview highlights the barriers to accessing mental health services in Canada and underscores recent federal funding commitments to enhance the availability of evidence-based, cost-effective solutions to improve access (5). The Common Statement of Principles on Shared Health Priorities agreement (10) is driving action across all jurisdictions in Canada to expand community-based mental health promotion programs and early interventions (5).

Nationally and provincially, there has been a pressing demand from mental health advocates for the implementation of adaptable, readily available, and cost-effective early intervention programs to bridge the gap in health services and provide a clinically and cost-effective rapid intervention (5, 11, 12). These initiatives should aim to reduce the emergency department visits and inpatient treatment while also addressing the prolonged wait times for individuals seeking psychiatric assistance (13). Such programs can be seamlessly integrated into the stepped models of mental health care, where individuals undergo prompt and thorough mental health assessments before being directed toward services tailored to their specific needs. The stepped care model emphasizes the provision of easily accessible early intervention programs, which offer the least restrictive and least costly interventions to the majority, thereby enhancing access to mental health services through more efficient resource allocation (14). This approach not only reduces wait times for clients but also helps curtail unnecessary utilization of high-cost mental health services such as inpatient care and emergency department visits (15). In response to the urgent call by stakeholders of mental health in the province, Nova Scotia Health (NSH) has been actively enhancing access to high-quality addiction and mental health services by implementing a range of services and technology-driven health initiatives (16) (17).

### 1.1 Rapid access and stabilization program

To further improve capacity and accessibility in mental health care, the province introduce an innovative mental health initiative the “Rapid Access and Stabilization Program” (RASP) as a pilot initiative. The RASP aims to provide expedited psychiatric consultation, treatment plan recommendations for primary care providers, and digital mental health supports for patients to reduce overall wait times for accessing community mental health services, decrease emergency department visits for mental health concerns, and minimize the need for inpatient psychiatric treatments. For the full year of the pilot, RASP human resources assigned to the program through re-purposing existing resources included three psychiatrists with each providing two days (0.4FTE) of assessments at the clinic and 0.6 FTE of admin assistance, and the program operated five days a week from 8.30 am to 4.30 pm. The psychiatric resource used to establish RASP was repurposed from community services as part of broader efforts to enhance clinical efficiency and operational excellence. This decision followed a detailed service model analysis, which concluded that reallocating a portion of psychiatric FTEs from certain community programs would not adversely affect the quality or volume of care those services provide. Each psychiatrist was scheduled to assess four new patients on the days that they worked at the RASP. To ensure operational efficiency and clinical governance, one of the RASP psychiatrists was designated the Clinical Academic Leader for the program, and the program was also assigned a program manager by the Mental Health and Addictions Program (MHAP) operational director. The RASP, a tier 3 model of care, was established and implemented at the end of April 2023 and situated in QE II Health Sciences Centre in Halifax.

Primary healthcare providers (PHP), which encompass nurse practitioners and general practitioners, including those who work in walk-in clinics, can refer patients to the RASP through the established central intake pathway, a centralized telephone intake service through a provincial toll-free number Monday to Friday and all week-day holidays 8:30 a.m. to 4:30 p.m. as well as Tuesday and Thursday evenings until 8:00 p.m. Anyone can self-refer to the Mental Health and Addictions Intake service or a service provider with consent can make a referral on their behalf and the individual will be called to participate in an Intake assessment). Additionally, psychiatrists involved in the program offer telephone consultations and assistance to PHPs as part of follow-up support for patients seen at the RASP.

Furthermore, patients receiving mental health care within the RASP can opt in to be enrolled in a Text4Support program which delivers primary mental health concern-specific and general wellbeing-related cognitive behavioral therapy-based daily supportive text messages (18). The Text4Support programs can tailor content based on problems related to anxiety, depression, psychosis, mood swings, stress and trauma, addiction, personality, and general well-being. After the psychiatric consultation, patients are given the chance to offer feedback on their experience with the RASP through either a paper-based or online satisfaction survey. An online survey link is sent to patient’s cell phones via text message following their appointment.

The satisfaction survey encompasses patients’ reactions to various aspects of their healthcare experience, evaluating both mental health services and providers subjectively (19). Surveying patient satisfaction serves multiple purposes, such as influencing healthcare utilization, predicting future health-related behaviors, and determining whether patients would recommend their mental healthcare provider to others (20, 21). Patients’ satisfaction is a useful measure in evaluating communication patterns, as patients offer valuable insights into their experiences, including clarity and helpfulness of information received, barriers to care, and physician interpersonal behavior (22, 23).

Despite the extensive literature on the subject, evaluating patient satisfaction remains challenging (24). Satisfaction comprises various dimensions, not all of which directly correlate with the actual quality of care a patient receives. Critiques of patient satisfaction ratings include their failure to consider that patient expectations are often shaped by prior healthcare experiences (25). However, ensuring high-quality care and treatment is a primary objective for both patients and healthcare providers. That is, healthcare organizations worldwide are facing mounting pressure to maintain quality of care amidst the rising demands for healthcare services.

The current study aims to explore the early impacts of the RASP on access to psychiatric consultations and wait times to see a psychiatrist after referral, comparing its outcomes to the standard care provided by Community Mental Health Clinics before the implementation of the RASP. Additionally, the study examines service users’ satisfaction with RASP, focusing on aspects such as access to care, wait times, overall experience, and areas for improvement. The ultimate goal is to leverage these insights to enhance patient-centered care, improve mental health outcomes, and optimize the delivery of mental health services to narrow the gap in psychiatry treatment for patients in Nova Scotia.

Specifically, this study seeks to address the following research questions:

1. How has RASP affected access to psychiatric consultations and wait times to see a psychiatrist after a PHP referral compared to the standard care before the RASP implementation?
2. What impact has RASP had on the overall number of new patients, regardless of referral source, who accessed psychiatric consultations?
3. What are patients’ perceptions of their overall experience and satisfaction with the RASP service, including aspects such as access to care, wait times, and quality of psychiatry consultation?

## 2 Method

### 2.1 Study design

This exploratory, descriptive study uses retrospective secondary data to compare patient access to psychiatric consultations in standard care and RASP. Data on psychiatric consultations from the two prior fiscal years (2021/2022 and 2022/2023) is compared to the data from the RASP’s first year of operation (2023/2024) to assess changes in psychiatric consultations. Wait time to see a psychiatrist was assessed using the median and 90th percentile of the time taken for patients to access a RASP psychiatrist after referrals were received by the central intake system during the first year of the program.

Patient satisfaction data were collected from the RASP satisfaction survey completed by patients between May 1st, 2023, and April 30th, 2024. Qualitative responses to open-ended survey questions were analyzed using content analysis to identify common themes and patterns, supported by verbatim quotes. This qualitative approach enabled the collection, organization, and categorization of participants’ experiences into different themes to determine what, how, when, or why a given aspect or reaction was given (in this case, the experiences of participants with RASP) (26).

### 2.2 Study setting and participants

Mental health care in Nova Scotia is publicly funded and delivered by NSH through the MHAP, which serves residents across four administrative zones: Central, Eastern, Western, and Northern. The RASP specifically serves patients within the Central Zone (CZ), located in Halifax, the provincial capital. The study on patient access to psychiatry consultations and wait times included all new RASP patients and those receiving psychiatry consultations through standard care. For the patient satisfaction component, only RASP patients who completed the satisfaction surveys were included.

### 2.3 Data collection and study procedures

Secondary data on the number of new patients accessing psychiatric consultations in the CZ/NS from 2021/2022 to 2023/2024 were extracted from NSH administrative databases. The RASP satisfaction survey (Appendix I) consisted of 19 questions, including 15 mandatory questions (one Likert scale and 14 multiple-choice) and four optional open-ended questions. The survey explored barriers to accessing the program, overall satisfaction, likelihood of returning for future assessments, and likelihood of recommending the program. Qualitative feedback specifically asked about barriers to accessing the service, which was displayed if the respondent indicated encountering a barrier in a previous multiple-choice question. The subsequent open-ended questions asked what patients liked best about the service and what changes they would suggest and invited additional comments about their overall experience with the service. The survey was administered either electronically via a REDCap (27) online link sent to service users via text message or in paper format completed at the clinic. Participants completing the paper survey were asked to submit it confidentially in a collection box before leaving the clinic. Research assistants were available to assist participants if needed.

### 2.4 Outcome measures

The primary outcome measure was the percentage change in the total number of new patients accessing psychiatric consultations in the CZ/NS. This included patients either directly referred by PHPs or transferred from mental health clinicians through standard care, comparing data from the two fiscal years before RASP implementation with the fiscal year following its implementation. Wait times for patients to see a RASP psychiatrist from the time their referral was received by central intake were measured by the monthly median and the 90th percentile wait times during the first year of RASP operation.

Secondary outcome measures included overall patient satisfaction, evaluated by the mean score on a scale from 1 (worst experience) to 10 (best experience), and the frequency of satisfaction levels based on a Likert scale (very satisfied, mostly satisfied, neutral, mostly dissatisfied, and very dissatisfied). Additional satisfaction outcomes included the frequency of responses regarding the likelihood of choosing RASP services again and recommending RASP to family or friends. Finally, satisfaction with various aspects of the psychiatric consultation, treatment plan development, dispositions, and outcomes were measured by the frequency of positive responses (**“**yes, definitely**”** and **“**yes, to some extent**”**) and negative responses (**“**no**”**). For the qualitative component, open-ended responses from the patient satisfaction survey were analyzed using the content analysis method to provide deeper insights into patient experiences, barriers to accessing the service, and suggestions for improvement.

### 2.5 Data analysis

Quantitative data were analyzed using descriptive statistics with SPSS version 28 for Windows, with results presented as numbers and percentages. Median and percentiles were used exclusively to analyze wait times.

On the other hand, the study employed a qualitative design to explore participants**’** experiences with the RASP. Data were collected through open-ended qualitative responses in a REDCap survey, which allowed participants to provide in-depth reflections on their experiences with RASP. An inductive content analysis approach, as guided by Elo, S., & Kyngäs, H. (2008) (28), was used to systematically analyze the data, allowing the development of themes and categories directly from the participants**’** responses. After data collection, the qualitative responses were exported from REDCap and manually analyzed. The analysis followed an open coding process by two researchers (MA and RD), wherein key phrases and concepts were identified across the responses. The two researchers then compared their codes, discussed areas of divergence, and reached consensus through an iterative review process. Although we did not calculate a formal inter-rater reliability coefficient, the use of dual coding, comparison, and consensus ensured analytic rigor and trustworthiness. These codes were grouped into subcategories, and through an iterative process, broader categories and overarching themes were formed that represented the core experiences of the participants. This approach was selected for its ability to explore participants**’** subjective experiences without predefined codes, providing flexibility in uncovering underlying patterns and meanings. All responses to the open-ended questions were included.

### 2.6 Ethical considerations

This study was conducted following the Declaration of Helsinki and ethical approval was obtained from the NSH Research Ethics Board (REB File **#**1028254). All RASP patients received an informed consent form as part of their intake package, which allowed investigators to access their health services utilization records upon signing. RASP patients were informed about the purpose of the satisfaction survey, its voluntary nature, and the anonymity and confidentiality of their responses. Completion of the survey was considered implied consent to participate in the evaluation of the RASP.

## 3 Results

### 3.1 Impact of RASP on access to psychiatric consultations

In the first year of operation (May 1st, 2023, to April 30th, 2024), 960 new patients gained access to psychiatric consultations at the RASP. The mean age of the patients was 40.12 years (SD ± 14.84 years), with a majority (64%) being female. Approximately 48% presented with depression and 25% with anxiety as their primary mental health concern. In the fiscal year 2023/2024 (**Table 1**), following the implementation of RASP, there was a notable increase in access to psychiatric consultations. A total of 959 unique patients were assessed through RASP during its first 11 months of operation, compared to only 36 patients in the standard care pathway. This represents a 1226.67% increase in the number of PHP-referred patients receiving psychiatric consultation through the community-based programs of the MHAP compared to the previous fiscal year. Overall, there was a 102% increase in unique patients accessing psychiatric consultations within both RASP and standard care services regardless of referral source, with 1971 patients in 2023/2024 compared to 976 patients in 2022/2023. This significant rise in access highlights the impact of RASP on enhancing mental health service availability within the CZ.

**Table 1:** Overall unique patients having access to a psychiatry consultation within all available services pre- and post-RASP implementation.

Service type	Fiscal Year (FY)	Unique primary care provider–referred patients who gained direct access to a psychiatric consultation in each service		Overall number of unique primary care provider–referred patients who gained direct access to a psychiatric consultation across both services.		Overall number of unique patients who received a psychiatric consultation following transfer of care from program mental health clinicians to a psychiatrist.		Total unique patients receiving psychiatric consultation—both those referred directly by primary care providers and those transferred from program mental health clinicians.	
		N	% change from previous FY	N	% change from previous FY	N	% change from previous FY	N	% change from previous FY
Standard care	2021/2022	94	–	94	–	994	–	1088	–
	2022/2023	75	-20.21%	75	-20.21%	901	-9.36%	976	-10.29%
	2023/2024	36	-52.00%	995*	+1226.67%	982	+8.99%	1971*	102.00%
RASP	2023/2024	959*	–			–	–		

^*^Fiscal year 2023/2024 runs from April 1st, 2023, to March 31^st^, 2024, which captures only 11 months of RASP data (May 1^st,^ 2023 to March 31^st,^ 2024). Data for the 2023/2024 fiscal year was adjusted by adding one one-month average for the 11 months of RASP data to represent the month of April 2023.

**Table 1** shows that in the first year following RASP implementation, the number of primary care provider–referred patients gaining direct access to a psychiatric consultation increased by 1,226.67%. Additionally, the total number of patients receiving a psychiatric consultation both those referred directly by primary care providers and those transferred from program mental health clinicians rose by 102%.

**Figure 1:**
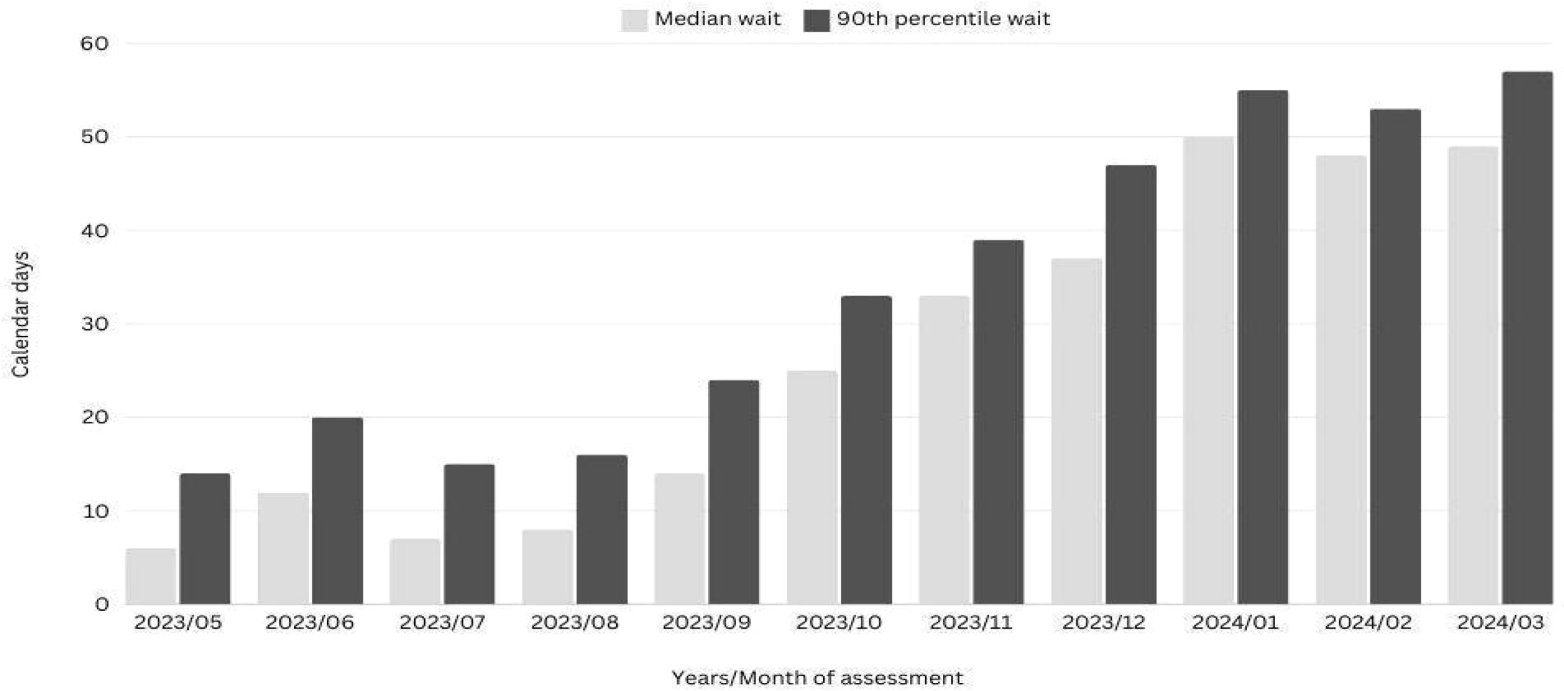
Median and 90th percentile wait time to see a Psychiatrist at RASP.

**Figure 1** illustrates the median and 90^th^ percentile wait times for access to a RASP psychiatrist from the time referrals are received by the MHAP central intake system. Data indicates that from May to September 2023, both the median and 90th percentile wait times for access to a psychiatric consultation within the RASP were under 30 days. The wait times increased to over 30 days from November 2023, with the median and 90th percentile wait times being 49 days and 57 days respectively as of the end of March 2024.

Data from the CMHAP policy unit indicates that as of March 2024, the median and 90^th^ percentile wait time for patients to receive an assessment from a mental health clinician within the CMHAP in the Central Zone of Nova Scotia was 48 days and 114 days, respectively. In addition, after the initial assessment from a mental health clinician, the median and 90^th^ percentile wait time for patients to begin treatment with a mental health clinician was 14 days and 26 days, respectively. Thus, as of March 2024, at least half of patients seeking mental health support were waiting more than 62 days to begin treatment with a mental health clinician who may subsequently refer those who needed psychiatric input for diagnostic clarification, medication consultation, or treatment plan recommendation to a psychiatrist. The majority of patients seen by psychiatrists in the community mental health program are internal transfers from mental health clinicians. For these patients, the wait-time from when they were referred or self-referred to Central Intake service to when they see the mental health clinician is tracked, but the wait-time for when they are seen by a psychiatrist is not currently tracked.

### 3.2 RASP patient satisfaction: quantitative results

**Figure 2:**
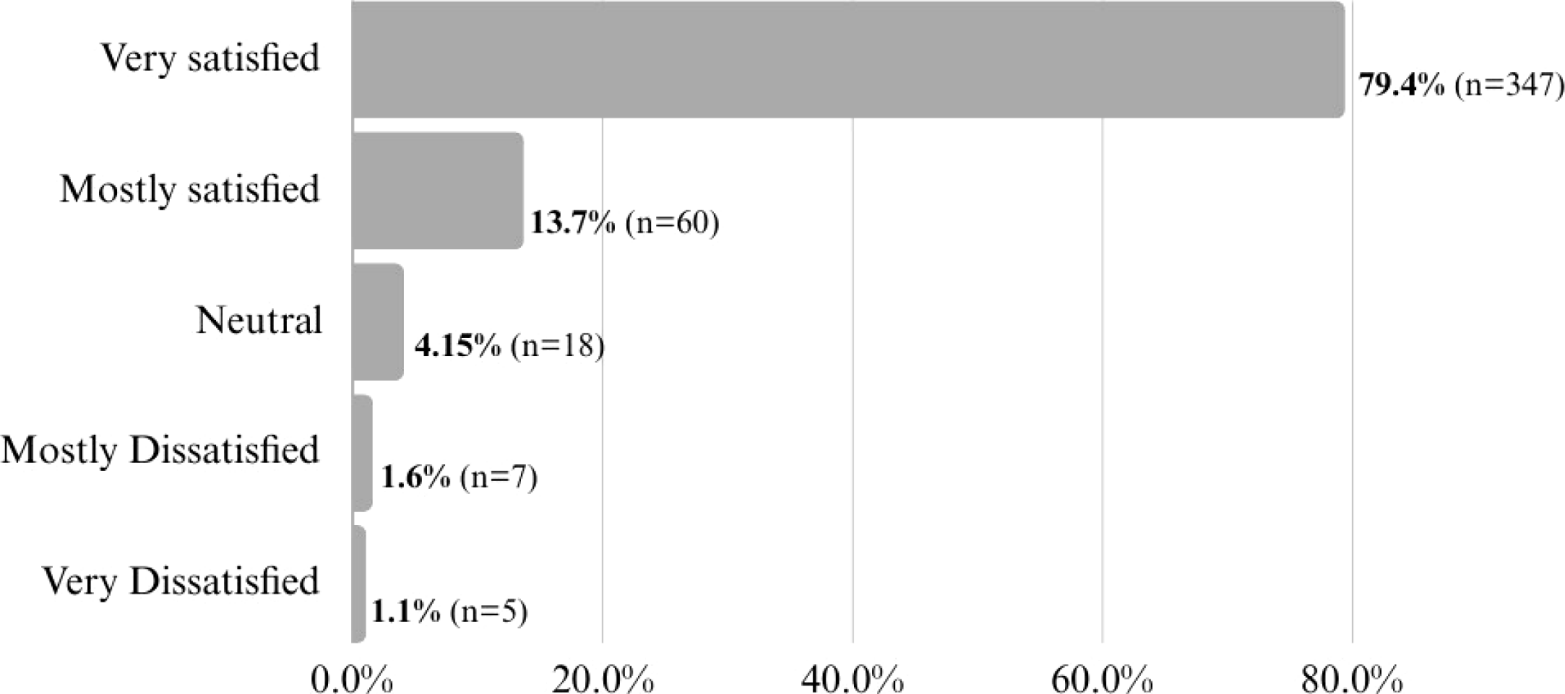
Overall level of satisfaction with the RASP.

In the first year of RASP operation, a total of 475 patients completed the online (REDCap) and paper-based satisfaction survey. **Figure 2** demonstrates that 93.1% of RASP service users reported being either “very satisfied” or “mostly satisfied” with the service provided. Only a small percentage expressed neutral or negative satisfaction levels. Similarly, when RASP patients were asked to rate their overall experience on a scale from 1 (worst possible experience) to 10 (best possible experience), the average satisfaction rating was 9.31 (SD = 1.42) based on 437 responses, indicating a high level of overall satisfaction with the service.

**Figure 3:**
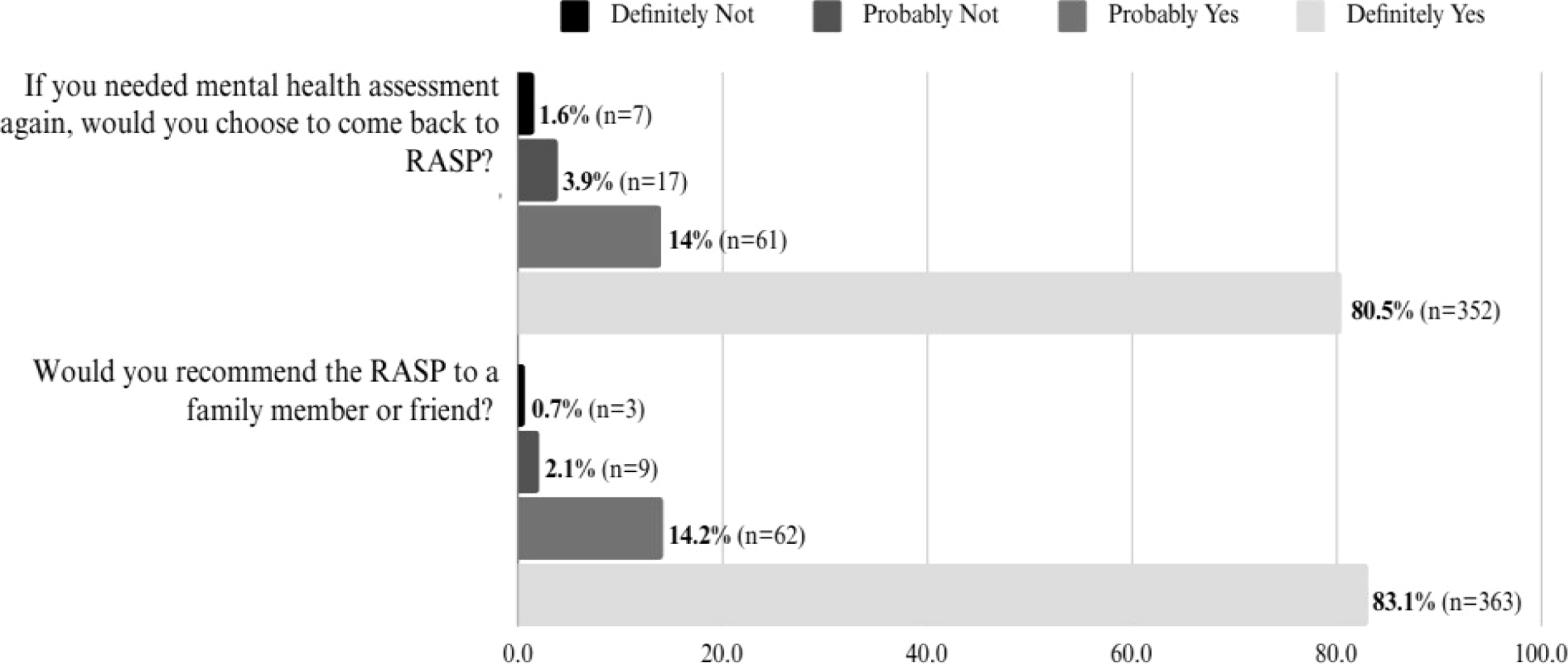
Participants feedback on choosing RASP or recommending the service.

When asked about their likelihood of choosing RASP services again, 94.5% of respondents indicated they would either “definitely” (80.5%) or “probably” (14.5%) return to RASP for a mental health assessment if needed. Similarly, when asked about recommending RASP to family and friends, 97.3% of respondents said they would “definitely” (83.1%) or “probably” (14.2%) recommend the service to others (**Figure 3**).

**Figure 4:**
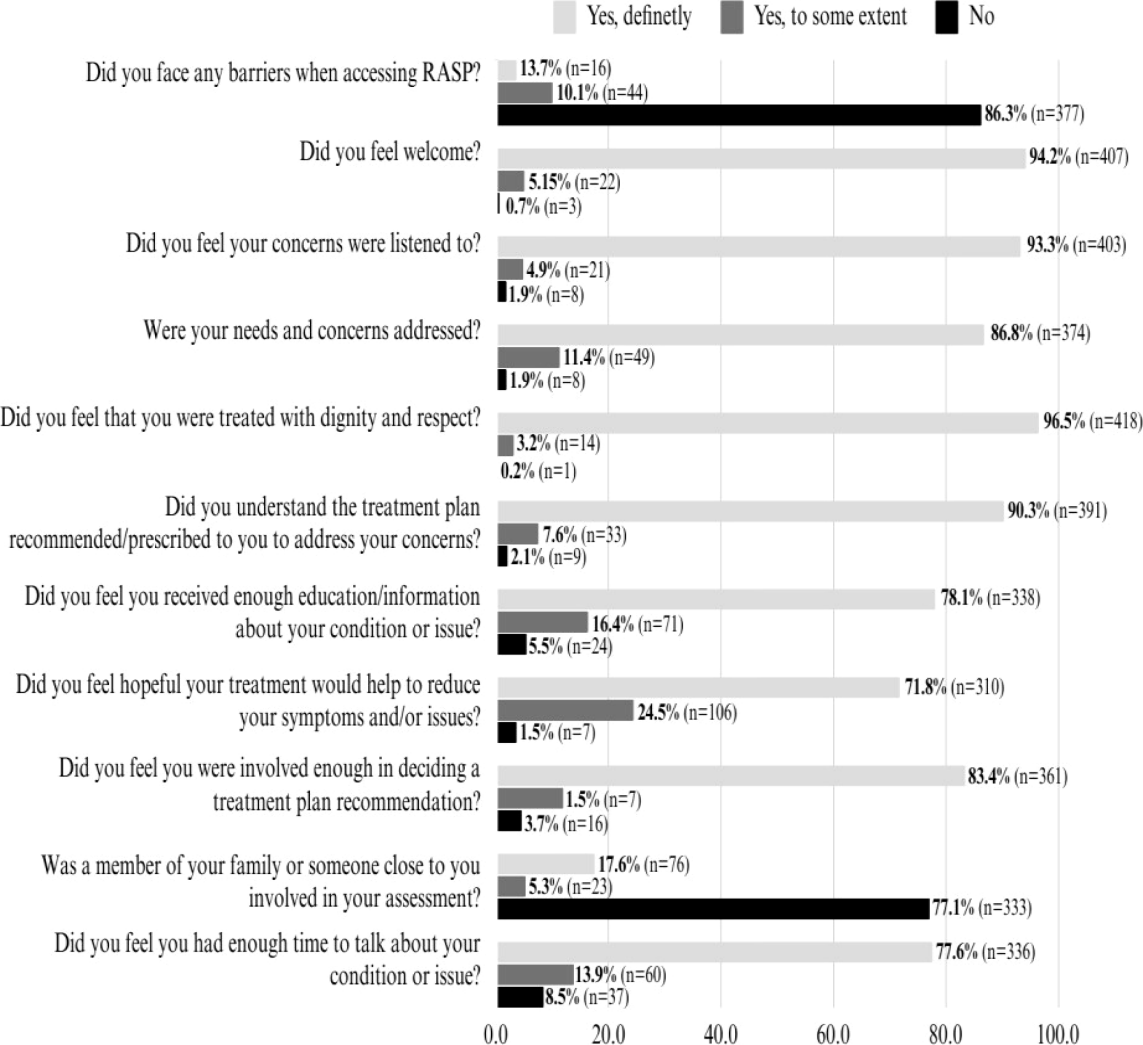
Participants feedback on satisfaction level on the service received at the RASP.

**Figure 4** demonstrates respondent’s satisfaction levels with various aspects of the service they received at the RASP. Concerning satisfaction related to how they were treated by the RASP team and psychiatrist, almost all respondents conveyed feeling welcomed (99.3%), listened to (98.2%), concerns being addressed (98.2%), and being treated with dignity (99.8%). Related to the treatment plans at the RASP, 97.9% of respondents indicated they at least to some extent understand the treatment plan recommended to address their concerns, and 96.3% reported they felt hopeful the treatment plan would help to reduce their symptoms. Overall, 95.4% of respondents indicated they were involved at least to some extent in deciding a treatment plan recommendation for their condition. However, only 22.9% of respondents indicated that a family member or caregiver was involved in their assessment at the RASP. Finally, 91.5% of respondents indicated they had enough time to talk about their condition or issue, and 94.5% of respondents indicated they received enough education/information about their condition or issue.

### 3.3 RASP patient satisfaction: qualitative results

Many service users provided qualitative responses to the four optional open-ended questions. The first open-ended question, displayed only to those who responded “yes, definitely” or “yes, to some extent” to the question about barriers to accessing the program, received 45 valid responses out of 60. Th remaining 15 responses were classified as invalid because they were blank, contained non-informative text (e.g., “N/A,” “none,” “no comment”). The analysis of these responses identified several key challenges (**Table 2**). The most frequently reported barriers were related to the referral process, including delays and difficulties with PHP referrals. Other notable barriers included challenges in accessing the RASP clinic, as well as administrative and personal obstacles. These findings highlight a range of logistical, administrative, and personal factors that hinder access to RASP services.

**Table 2:** Patient-reported barriers to accessing RASP services.

Theme	Sub-theme	Number of comments	Representative quote
Referral Process	Delays	12	“The time from referral to receiving the service was a barrier. It took about 5 weeks or more as my family doctor was very busy.”
	Challenges with PHP referral	8	“My family doctor required me to meet with her in order to refer me to the program. This introduced delays and emotional difficulty.”
Accessing RASP	Location	9	“Had a hard time finding the new relocation office and feeling a little unheard.”
	Parking and Transportation Issues	6	“Traffic at a standstill in several areas in the city, very little available parking in the hospital parkade.”
	Accessibility	3	“Phone is mandatory, even with a hearing deficiency.”
Administrative barriers	Communication	4	“They could not find my referral or my appointment for a week until they re-faxed a new one.”
Personal barriers	Social anxiety	3	“My own anxiety made it difficult, but access to RASP was incredibly amazing and fast.”
Total		45	

For the question regarding what patients liked most, 451 out of 475 survey respondents provided an answer. After excluding six incomplete responses and two stating “nothing specific,” 442 were included in the content analysis. Five major themes and eleven sub-themes emerged from the analysis (**Table 3**). The majority of comments were related to the short wait times, with 77 respondents expressing satisfaction with the quick access to appointments. Compassion and empathy from staff and psychiatrists were also highly appreciated, as 73 respondents highlighted the kindness, understanding, and compassionate approach. Another 73 patients emphasized the feeling of being heard. Additionally, 54 comments appreciated the clarity of the treatment plans, while 47 respondents praised the expertise and knowledge of the psychiatrists. Other notable themes included thoroughness of the assessment, accurate diagnosis, and a welcoming atmosphere.

**Table 3:** Key features of RASP services most valued by patients.

Theme	Sub-theme	Number of comments	Representative quote
Rapid Access	Fast appointment scheduling	7	“Everything was arranged for me, just had to show up when the intake person told me to.”
	Short wait time	77	“How quick it was to speak to someone. The intake personnel were amazing and quick appointment time. I am used to year waitlists.”
Professionalism and expertise	Thoroughness of assessment	24	“The psychiatric assessment was thorough, and the doctor asked detailed questions to get to the root of the issue. I was also able to bring my spouse, who can sometimes explain my symptoms better than I can. The doctor also read a good chunk of my medical file, which definitely told a story. I was very happy with the service I received!”
	Knowledgeable psychiatrists	47	“How knowledgeable the doctor was of my particular situation. Very understanding and helpful.”
	Comprehensive diagnosis	18	“The diagnosis was accurate and the recommendation of an increase in my medication was appropriate, and I agreed with it.”
Compassion and empathy	Kindness, understanding, and compassionate staff and psychiatrists	73	“Everyone I interacted with was very kind, welcoming, and understanding.”
	Feeling heard	73	“I feel that someone really listened to how I felt and wanted to help me.”
Comfort and welcoming environment	A comfortable and welcoming atmosphere	13	“I felt welcomed and comfortable/safe.”
	Open and non- judgmental environment	20	“Open floor, with no judgement, caring professionals who were open and truthful.”
Effectiveness of care	Accurate recommendation options	26	“I was listened to and provided with multiple options of support and information about my diagnosis. Very informative and thorough.”
	Treatment plan clarity	54	“I have been given a clear plan to follow moving forward. I understand and agree with this plan.”
	Felt on the way to recovery	10	“I am thankful things will be moving forward. There is always hope.”
Total		442	

Input on suggested changes to the RASP services was provided by 301 patients. However, 58.13% (n=175) of the responses were “nothing,” “N/A,” or “none.” Three incomplete responses were excluded, leaving 123 responses for analysis (**Table 4**). The majority of comments were related to the desire for follow-up appointments (18.25%, n=23), where patients expressed the need for automatic follow-up sessions instead of requiring a new referral. Another significant portion of the feedback focused on improving communication (15.07%, n=19), with suggestions such as providing more consistent directions to the clinic and informing patients in advance about what to expect during their visit. Other notable themes included enhancing coordination of care (12.7%, n=16), requests for longer appointments (11.1%, n=14), and the desire for expanded services to benefit more patients. These findings reflect patient desires for improved service delivery, enhanced communication, and better coordination of care within RASP.

**Table 4:** Patient-identified areas for improvement in RASP.

Theme	Sub-theme	Number of comments	Representative quote
Service improvements	Speeding access to the service	6	“Making access easier, it took too long to get access.”
	Expanding the service	14	“This sort of stuff should be given to everyone. Everyone would benefit from psychological or psychiatric help.”
	Improving coordination of care	16	“The need to rely on my family doctor for follow-up. My family doctor has persistently shown a lack of interest in helping me with any mental health concerns, and in this province, there are significant barriers to changing family doctors.”
	Improving clinic environment	11	“The office could use a bit more inviting furniture/decor.”
	Enhancing communication	19	“Tell people about the giant questionnaire in advance. I was early had time, other may not.” “More consistent directions to the location. A preliminary communication informing you what to expect when you arrive and have the session.”
	Psychiatrist approach	14	“I would like some other options for treatment besides CBT.”
Improve inclusiveness and accessibility	3	“More inclusive, less intimidating/threatening space.”
Program awareness and resources	Program promotion	3	“I wasn’t told it was called the rapid access program or the details. provided an apt. the program itself was not discussed. I googled it once I received the survey link.”
Patients wishing	Follow-up appointments	23	“I wish there was an automatic follow-up with the psychiatrist instead of having to go through the referral process again.”
	Longer appointments	14	“Appointments don’t need to be rushed. Too little time.”
Total		123	

Finally, 211 patients included additional comments in response to the last question. Among them, 67 (31.75%) stated they had no additional comments. One response was excluded due to incompletion, resulting in 143 responses being analyzed (**Table 5**). The majority of comments were related to positive service experience, with 45 comments highlighting overall satisfaction, such as praise for the service and timely care, and positive feedback on the psychiatrist’s approach and treatment plan. Gratitude and hopefulness were expressed in 43 comments, emphasizing the value of the service in preventing crises. On the other hand, there were 16 negative comments about overall dissatisfaction, dissatisfaction with the treatment plan, clinic location, and parking issues. Suggestions for improvement included enhancing communication, expanding the program, improving the clinic environment, providing patient education materials, and adding follow-up appointments.

**Table 5:** Patient-reported additional comments on the RASP satisfaction survey.

Theme	Sub-theme	Number of comments	Representative quote
Service experience	Overall satisfaction with the service	45	“All the doctors/nurse practitioners I’ve interacted with/through this service have been incredible. I hope the team understands that their dedication and genuine investment in patient wellness does not go unnoticed.”
			“I have been dealing with the mental health system for a long time in Nova Scotia, this is the first time I’ve ever been seen in a timely manner and connected with the person who would be helping me. I found solutions and hope for the first time and I think that is incredibly valuable. I hope others feel the same with this program!”
	Satisfaction with the rapid access to the service	5	“It was indeed rapid access which I appreciated as waiting for help is difficult.”
	Dissatisfaction regarding the duration of the appointment	3	“The rapid-fire pace of the questioning of me was too fast and she. Didn’t give me enough time in between them to give full or accurate responses.”
	Positive feedback on the psychiatrist’s approach/treatment plan	14	“I was impressed that the Doc explained everything that she was doing in detail, including writing notes during the session, rather than waiting until afterward like most doctors. I felt confident that she had everything down and would not have to rely on her memory alone.”
Positive personal feedback	Gratitude/hopefulness	43	“Thank you for this service, it prevented me from seeking crisis care at the ER.” “Thank you, not sure I would have been able to get help without it.” “Thank you so much from the bottom of my heart.”
Negative personal feedback	Overall dissatisfaction	7	“I was very disappointed with the care and advice I received. Telling me I was kind over and over again did not help me in any way.”
	Dissatisfaction with treatment plan	2	“I was there because I have A.D.D. and was hoping to get a proper med. for it … perhaps Adderall. he was focusing more about the fact I am a hoarder….I am working on decluttering myself it was the focusing problem I was there. For. He never got back to my doctor, so I just put it down to “at least I tried.”. I am cleaning out a storage and if I can’t focus, I will just buy an energy drink!”
	Clinic location and parking issues	7	“Super hard to find the clinic in the hospital.” “There is construction going on in the parkade. Plan a few extra minutes.”
Suggestions for improvement	Clinic environment	2	“More comfortable chairs in the clinic would send the message that patients are invited to feel comfortable and safe in the space.”
	Enhance communication	6	“Perhaps more clarity that this is a one-off appointment with the potential for infrequent follow-up, it is not matching with an ongoing psychiatrist that you can see on a weekly/monthly basis.” “I would have loved for a dedicated website (or webpage on another NS health website) detailing how exactly to go about self-referring. Maybe this already exists, but I couldn’t find it.”
	Patient education materials	2	“Some handouts with info about ADHD, depression anxiety, etc. would be nice.”
	Program expansion	5	“Expand the program because it is an important tool in helping people with mental illness. It gives hope.” “Give them more funding, this is a fantastic service.”
	Follow-up appointments	2	“Patient follow-up visits with the psychiatrist at months 2, 5, and 8 following the initial assessment would be wise … to update the patients’ mental health situation and to monitor their progress (if any). I am hopeful that I can see my psychiatrist at least one more time to see where my progression lies.”

## 4 Discussions

The increasing demand for mental healthcare services and barriers to accessing these services contributes to worsening mental health conditions, high acute health services utilization and healthcare costs (5). Improving access to community based mental health early intervention services would improve the mental health of the population, reduce utilization of acute care services, and reduce overall healthcare cost (5).

### 4.1 The RASP model and increased access to psychiatric consultation

The findings from this study demonstrate a significant increase in access to psychiatric consultations following the implementation of the rapid access and stabilization program, highlighting its substantial impact on psychiatric services in the central zone of Nova Scotia. In the first 11 months of operation, the RASP facilitated access for 959 unique patients, in stark contrast to the 36 patients seen under the standard care pathway during the same period in addition to its ongoing caseload of patients already in follow-up care. The over 1226% increase underscores the effectiveness of RASP in addressing the demand for psychiatric consultations, a critical step in improving mental health outcomes. It should be noted that in community MHA clinics, while psychiatric consults occur primarily for urgent patient referrals from the MHAP Central Intake service, the psychiatrists in the clinics provide ongoing follow-up appointments and care for patients with severe and persistent mental health problems, which diminishes the capacity for these psychiatrists to conduct routine new psychiatric consultations for PHP referred patients. This diminished capacity of the MHA clinics to provide consultation services for PHP referred patients, coupled with the demonstrated success of the RASP to increase access to psychiatric consultation for PHP referred patients justifies the creation of the RASP to address the highlighted treatment gap.

Furthermore, the overall 102% increase in unique patients accessing psychiatric consultations in the CZ during the fiscal year 2023/2024, compared to the previous fiscal year (2022/2023), provides further evidence of the positive impact of RASP. This exponential increase demonstrates that RASP is not only mitigating the bottlenecks in standard care pathways but also attracting new patients who might have faced delays or barriers in accessing psychiatric care previously. This finding is extremely important given that increased access to mental healthcare consultations is a critical element of mental health service delivery and that timely intervention in mental healthcare is associated with improved outcomes, reduced symptom severity, and lower rates of hospitalization (29, 30). By significantly improving access, RASP is contributing to a more responsive mental health care system. The reduction in patient numbers within the standard care pathway (from 75 patients in 2022/2023 to 36 in 2023/2024) is worth mentioning and likely reflects the transition of patients from standard care to the RASP model. This shift implies that RASP is absorbing a significant portion of the demand for psychiatric consultations, thereby reducing the pressure on traditional pathways. Thus, the reallocation did not diminish psychiatric consultation capacity for other service areas and that the RASP model in fact alleviated existing bottlenecks in PHP-referred access by creating dedicated consultation infrastructure that previously did not exist. This transfer of care aligns with global trends toward streamlined, patient-centered approaches in mental health services to reduce wait times and improve access to care (7). Furthermore, the ability to manage nearly 1,000 patients in under a year of its implementation signifies RASP’s scalability and potential as a sustainable solution for increasing access to mental health care consultation within CMHAP.

### 4.2 Comparison of RASP and CMHP service user profiles

In a related analysis comparing individuals accessing support through RASP with those receiving care from the Community Mental Health Program (CMHP). Participants from the CMHP were individuals enrolled in a clinical trial evaluating the effectiveness of Text4Support, a CBT-informed supportive text-messaging program designed to enhance mental health care for patients receiving or transitioning from psychiatric services in Nova Scotia (18). Notable sociodemographic and clinical differences were observed. Using chi-square tests and independent t-tests, the study identified significant variation between the two groups. RASP participants were generally older (M = 40.10 vs. 34.52 years) and demonstrated greater socioeconomic stability, as reflected in higher rates of employment (55.3% vs. 47.9%) and homeownership (36.5% vs. 17.7%). Conversely, CMHP participants experienced higher unemployment (25.7% vs. 16.5%) and were more likely to fall within lower income brackets, with nearly half (47.5%) earning less than CAD 29,590 annually compared with 30.3% among the RASP cohort. Clinical profiles also differed substantially. Depression was more commonly reported among RASP users (48.2% vs. 19.3%), whereas CMHP participants exhibited higher rates of psychosis (10.6% vs. 2.5%) and substance use disorders (7.8% vs. 1.9%). Symptom severity scores further highlighted these distinctions: RASP participants reported higher levels of anxiety (GAD-7: M = 14.17 vs. 11.81) and depressive symptoms (PHQ-9: M = 16.62 vs. 14.20), alongside lower resilience (BRS: M = 2.47 vs. 2.77). In contrast, CMHP participants reported more adverse childhood experiences (ACE: M = 3.92 vs. 3.16) and lower suicidal intent, with 81.4% indicating no intention to act compared to 99.4% within the RASP group (31).

Together, these findings suggest that RASP and CMHP may be serving distinct populations with differing sociodemographic contexts, clinical needs, and risk profiles an important consideration when interpreting outcomes and planning service delivery across the continuum of mental health care.

### 4.3 The RASP model and reduced wait times

The relatively short wait times for psychiatric consultation within the RASP in its initial months of implementation (under 30 days for the median and 90th percentile) implies that this rapid access model is effectively addressing one of the major barriers in mental healthcare services: timely access. RASP’s ability to provide psychiatric consultation within a shorter time frame likely contributes to faster diagnostic clarification, earlier medication adjustments, and prompt treatment planning. This advantage is especially essential given that psychiatric conditions often require immediate action to stabilize symptoms and prevent deterioration (32, 33). By providing psychiatric services more rapidly than traditional care pathways, RASP may be better positioned to mitigate patient distress and improve clinical outcomes.

These findings align with existing literature, which highlights how crisis resolution teams and rapid access models can effectively reduce hospital admissions and emergency department visits by ensuring the provision of timely and effective mental health care (34). By improving the triage process and leading to direct psychiatric consultation, RASP ensures that patients receive timely and appropriate mental healthcare, thereby reducing the need for emergency interventions and or admissions. This result is consistent with an earlier report that discussed the economic impact of mental health services and highlighted the cost savings associated with early intervention to mental health issues and rapid access programs (6). The subsequent increase in wait times by the end of March 2024, however, demonstrates the demand for RASP services has grown, potentially outpacing capacity. This problem often occurs in programs that initially reduce access barriers, as success often leads to increased referrals and higher demand for services (35). The steady increase in wait times, as observed in the study, may reflect the growing popularity of the service among PHPs and their patients and the need for further scaling of the RASP model, either through additional staffing or expanded resources to maintain timely access as the program matures. For example, for the full year under report, there were three part-time psychiatrists, each providing only two days of psychiatric consultation services with the program for a total of 1.2 full-time equivalence (FTE). Increasing the psychiatry complement to 4.0 FTE and extending the operating hours to 8 pm will create the capacity to meet the growing need for the service for Nova Scotia’s one million population. Notwithstanding the growing wait times, given it takes an average of 62 days for patients to access a mental health clinician for treatment in the Central zone, who could make a referral to a psychiatrist within the CMHAP at a future date, it is reasonable to conclude that patients are still having access to a psychiatrist faster than if they were to be seen through the standard care pathways.

### 4.4 Patient experience/satisfaction with RASP: quantitative and qualitative outcomes

The importance of patients’ experiences is increasingly acknowledged as a fundamental aspect of achieving excellence in healthcare. Satisfaction serves as a significant outcome measure in assessing and enhancing healthcare quality for mentally ill patients. Despite the acknowledged value, there remains a limited number of satisfaction surveys focused on mental health, particularly in cases of severe mental illnesses, although their importance is no longer a subject of debate. This study offers valuable perspectives on the initial outcomes and efficiency of the Nova Scotia Transformative RASP in meeting the required mental health needs of patients within the catchment area. Through the evaluation of patient satisfaction with various aspects of the program, including access, communication, and overall experience, this study sheds light on the strengths and areas for improvement within the RASP model of care.

The findings from the data on the patients’ experience questionnaire demonstrate positive experiences with RASP, evidenced by a mean satisfaction rating of 9.31 and high percentages of respondents feeling warmly received, listened to and treated with dignity. However, there were a few areas for improvement, particularly program location and access to parking, which were noted. Addressing these challenges is crucial not only for the well-being of persons with mental health problems but also for enhancing the overall quality and efficiency of the mental healthcare system.

Furthermore, the program’s success in reducing common barriers to access, such as long wait times, is reflected in the findings that 86.3% of participants experienced no barriers to accessing the RASP. This reduction in barriers not only reduces the chances of patients delaying care until they require emergency services but also contributes to lowering the high overall healthcare costs by reducing mental health-related ED visits and providing timely psychiatric consultation and follow-up care, thus preventing the escalation of mental health crises that often lead to more costly emergency interventions. These findings align with a review that identified common barriers to accessing mental health care and how addressing these barriers improves mental health outcomes and reduces the need for emergency care (29). As observed from the qualitative findings from the study, one significant strength of the RASP, as identified by participants, is its ability to provide timely access to mental health care services. Thus, respondents appreciated the efficiency in scheduling appointments, especially during times of crisis or urgent need. This aspect of the program is particularly important given the significant barriers associated with accessing mental health care services, including long wait times and limited availability of appointments. This finding is consistent with literature that supports the importance of timely access, showing that quick response times are linked to improved patient outcomes and higher satisfaction levels (36). Additionally, the compassionate and knowledgeable nature of the psychiatrists and staff members associated with the RASP clinic was another key strength emphasized by the respondents. Feeling heard, understood, and supported by clinicians who demonstrate expertise in mental health care is essential for building trust and rapport between patients and providers. The emphasis on transparent communication and the thoroughness of the intake process were also valued by patients seeking guidance on their mental health journey. Consistent with literature and in various contexts, there is consistent evidence highlighting the significant role of the patient-practitioner relationship in enhancing satisfaction levels. Extensive research confirms that patients value a tailored approach and empathetic demeanor. They desire a physician who actively listens, offers informative guidance, and emphasizes collaborative decision-making (37). While the overall satisfaction with the RASP was high, participants also identified areas for improvement. About one in ten patients expressed a desire for longer appointment durations to allow for more comprehensive discussions about their mental health concerns. This is a common issue in mental health care, where the complexity of patient needs often requires more time than standard appointment slots allow. Research indicates that longer appointment times can lead to better patient outcomes and higher satisfaction as they enable more thorough assessments and stronger therapeutic alliances (38, 39). To meet this requirement within the current RASP structure, the addition of mental health clinicians to the RASP program who will provide support and guidance to patients after the psychiatric assessments, and coordinating patients’ care, including connecting patients with community supports or transfers of care to other providers within the MHAP, when necessary, will allow the RASP psychiatrist to utilize the full appointment times for assessments and treatment plan formulation. There were also suggestions for easier access to follow-up appointments to ensure continuity of care and ongoing support beyond the initial assessment. This concern is crucial since consistency in follow- up care is critical for maintaining treatment progress and addressing any emerging issues promptly. Studies have shown that regular follow-up appointments are essential for effective management of mental health conditions, reducing the likelihood of relapse, and improving long-term outcomes (40, 41). However, despite patients desire for follow-up underscores an important service gap in the broader system, addressing this gap likely requires solutions outside the RASP model such as improved transitions to community mental health services rather than restructuring RASP itself. In addition, because patients are referred back to their primary care providers after their RASP appointment, they retain the opportunity to receive ongoing follow-up through their usual care provider, which aligns with the program’s design as a rapid-access, consultation-focused service.

Furthermore, challenges related to locating the clinic and navigating the facility were noted by some patients, highlighting the importance of enhancing accessibility measures and communication to facilitate ease of access for individuals seeking mental health support.

## 5 Strengths and limitations

This study has several notable strengths. First, it employed a mixed-methods approach, combining quantitative analysis of access metrics from administrative data with qualitative insights from a patient satisfaction survey. This allowed for a more comprehensive understanding of the impact of RASP on both objective measures of access and subjective patient experiences. Second, the study evaluated a novel rapid access model in a real-world setting, providing valuable insights into the feasibility and effectiveness of implementing such models in mental health service delivery. Third, the focus on increasing access to psychiatric care for patients referred by primary care providers addresses a critical gap in the mental health system and aligns with the principles of stepped care models. Finally, the inclusion of patient perspectives through the satisfaction survey emphasizes the importance of person-centered care and highlights areas for improvement in RASP delivery. However, the study has some limitations that should be considered when interpreting the findings. The pre-post design without a control group limits the ability to draw causal conclusions about the impact of RASP on access outcomes. It is possible that other factors, such as changes in referral patterns or system capacity, could have influenced the observed changes in access metrics. Additionally, the use of administrative data may have limitations in terms of data quality and completeness. The patient satisfaction survey, while providing valuable qualitative feedback, used a convenient sample and cross-sectional design, which may limit the representativeness and generalizability of the findings.

The study was also conducted at a single site in one zone of Nova Scotia, so the results may not be generalizable to other settings with different patient populations or system characteristics. Future research should focus on longitudinal outcomes associated with RASP, such as patient satisfaction, symptom improvement, overall mental health outcomes, health services utilization and cost effectiveness. Moreover, studies comparing patient outcomes between those receiving care under RASP and traditional pathways would provide valuable insights into the program’s efficacy beyond access alone. Finally, exploring the scalability of RASP in other health zones or different populations could help determine its potential for broader implementation.

## 6 Conclusions

In conclusion, this mixed-methods evaluation provides initial evidence supporting the Rapid Access and Stabilization Program (RASP) as a promising model for increasing timely access to psychiatric care and providing patient-centered services in the Central Zone of Nova Scotia. The finding of substantial increases in patients receiving psychiatric consultations, particularly those referred by primary care, after RASP implementation addresses a critical need to improve access to mental health care. Furthermore, the high patient satisfaction ratings underscore the program’s acceptability and ability to deliver compassionate, person-centered care. While the study has limitations inherent to the pre-post design without a control group and the generalizability of findings from a single site, the results merit further implementation and evaluation of RASP in other regions.

## Data Availability

The raw data supporting the conclusions of this article will be made available by the authors, without undue reservation.
